# Prognostic significance of VEGF and components of the plasminogen activator system in endometrial cancer

**DOI:** 10.1007/s00432-020-03225-7

**Published:** 2020-05-11

**Authors:** Karin Abbink, Petra L. M. Zusterzeel, Anneke Geurts-Moespot, Rob van der Steen, Paul. N. Span, Fred C. G. J. Sweep

**Affiliations:** 1 Department Obstetrics and Gynecology, Radboud University Medical Centre, Geert Grooteplein 10, P.O. Box 9101, 6500 HB Nijmegen, The Netherlands; 2 Department of Laboratory Medicine, Radboud University Medical Centre, Nijmegen, The Netherlands; 3 Radiotherapy and OncoImmunology Laboratory, Department of Radiation Oncology, Radboud University Medical Centre, Nijmegen, The Netherlands

**Keywords:** Endometrial cancer, Prognostic factor, Plasminogen activator system, Vascular endothelial growth factor

## Abstract

**Objective:**

The plasminogen activator system (PAS) and vascular endothelial growth factor (VEGF) are important in the carcinogenesis and play a key role in cancer invasion and mediating metastasis of carcinomas. The aim of the study was to evaluate the correlation of serum levels of VEGF and components of the PAS with clinicopathological risk factors and outcome in patients with endometrial cancer (EC).

**Methods:**

Preoperative blood was collected from 173 patients treated for EC between 1999 and 2009. Serum concentrations of VEGF, urokinase plasminogen activator (uPA) tissue plasminogen activator (tPA), plasminogen activator inhibitor type-1 (PAI-1) and -2 (PAI-2) were assessed by enzyme-linked immunosorbent assays (ELISA).

**Results:**

Serum levels of VEGF and components of the PAS were significantly associated with stage of the disease, tumor histology, tumor grade, myometrial invasion (MI), presence of lymphovascular space invasion (LVSI) and lymph node metastases (LNM). Preoperative serum levels of PAI-1 and -2 and tPA were higher in patients who experienced a recurrence than in patients who remained disease free (*p* < 0.01). PAI-1 and -2 and tPA were significantly independent prognostic factors for DFS with a HR of 3.85 (95% CI 1.84–8.07), 3.90 (95% CI 1.75–8.66) and 2.53 (95% CI 1.16–5.55), respectively. PAI-1 and tPA turned out to be independent prognostic factors for OS, with a HR of 2.09 (95% CI 1.08–4.05) and 2.16 (95% CI 1.06–4.44), respectively.

**Conclusion:**

Serum levels of VEGF and components of the PAS at primary diagnosis were associated with well-known clinicopathological risk factors such as; FIGO stage, tumor histology, tumor grade, MI, LVSI and LNM. High concentrations of PAI-1 and-2 and tPA are independent factors for poor prognosis in patients with endometrial cancer.

## Introduction

In general, endometrial cancer (EC) causes early symptoms such as postmenopausal bleeding. Most women are diagnosed at an early stage and have a favorable prognosis. The overall five-year survival in endometrial cancer is 74–91%. (Creutzberg et al. 2003) About 13–17% of endometrial cancer patients will develop recurrent disease mostly within 3 years after primary treatment (Rauh-Hain and Carmen 2010; Testa et al. 2014). Prediction of the clinical behavior of endometrial carcinomas is important to prevent under and overtreatment, but this is still challenging in patients diagnosed with EC.

To date, patients with EC are categorized into risk groups (low, intermediate, and high) based on known prognostic factors such as; depth of myometrial invasion (MI), differentiation grade, tumor histology and lymphovascular space invasion (LVSI). Recommendations for adjuvant treatment (radiation and/or chemotherapy) are mainly based on these prognostic factors (Colombo et al. 2013). However, a part of the patients considered as low-risk will experience a recurrence, whereas a part of the patients considered as high-risk will not (Salvesen et al. 2012). In addition to histological prognostic factors several immunohistochemical and genetic markers have been described which could improve the categorization of these risk groups. The immunohistochemical loss of ER and PR expression is associated with lymph node metastases and disease recurrences (Trovik et al. 1990). More recently, expression of the L1 cell adhesion molecule (LCAM) seems to be one of the most powerful markers associated with a poor outcome in EC patients (Geels et al. 2016; Bosse et al. 2014).

To improve patients care we need to reevaluate existing markers, discover and validate new markers, and study the combined value of existing and new markers. To date, biomarkers are not routinely used in clinical practice and diagnostic work-up of patients with EC. Biomarkers such as components of the plasminogen activator system (PAS) and vascular endothelial growth factor (VEGF) which already proved their utility in other cancers could play a role in the risk stratification and individualization of EC treatment.

VEGF is important in the carcinogenesis since it is responsible for tumor growth and is also involved in the metastatic process. Angiogenesis is essential for tumor proliferation, progression and the formation of metastasis (Folkman 1995). Angiogenesis is the result of a balance between actions of pro- and anti-angiogenetic factors (Poon et al. 2001). Pro-angiogenetic factors include; VEGF and proteases (plasminogen activators, matrix metalloproteinases), which degradate extracellular components (collagens, laminins, proteoglycans) and others (Poon et al. 2001). VEGF is one of the most important pro-angiogenetic factors (Poon et al. 2001; Yokoyama et al. 2000). It plays an important role in endothelial cell proliferation and increases vascular permeability of tumor-associated blood vessels. It promotes the extravasation of proteins from tumor vessels leading to a fibrin matrix that makes invasion of stroma cells into a developing tumor possible (Poon et al. 2001; Rogers et al. 2009). To date, literature concerning serum levels of VEGF in EC patients is scarce, with conflicting results.

Cancer progression and especially tumor invasion and metastasis are dependent on the actions of various protease systems. An important protease system is the PAS, which not only controls the intravascular fibrin deposition but also participates in a variety of physiological and pathological processes such as tumor growth, invasion and metastasis (McMahon and Kwaan 2008). PAS consists of urokinase plasminogen activator (uPA), tissue plasminogen activator (tPA and plasminogen inhibitors (PAI-1 and -2) (Duffy and Duggan 2004; Andreasen et al. 1997). Plasminogen activators stimulate the conversion of plasminogen into plasmin, which consequently leads to disruption of the extracellular matrix (ECM), degradation of basement membranes, interruption of connective tissue and vascular and lymphatic spaces.

Previous studies showed the prognostic value of tumor tissue levels of uPA, tPA, PAI-1 and -2, and VEGF in predicting metastases, recurrence, therapy response, and survival of patients with various tumors types, such as breast-, lung-, and cervical cancer (Poon et al. 2001; Duffy and Duggan 2004; Witte et al. 1999a; Shaarawy and El-Sharkawy 2001; Saarelainen et al. 2014; Zusterzeel et al. 2009; Harbeck et al. 1990).

Breast cancer is the first and most extensively studied malignancy where uPA and PAI-1 are incorporated in clinical practice (Harbeck et al. 1990; Duffy 1996; Duffy et al. 1990, 2014; Schmitt et al. 2010). Several guidelines, such as European Organisation for Research and Treatment of Cancer (EORTC), and the American Society of Clinical Oncology (ASCO) recommend the invasion and metastasis markers uPA and PAI-1 for risk assessment and treatment decision in node-negative (N0) breast cancer patients (Harbeck et al. 1990, 2002; Schmitt et al. 2010; Foekens et al. 2000).

However, to date studies in EC concerning components of the PAS and VEGF are limited and results are conflicting.

As outlined above, there is an urgent need for additional markers for endometrial risk classification and individualization of treatment. Therefore, the aim of this current study was to evaluate the correlation of the serum biomarkers PAS and VEGF with clinicopathological risk factors and survival.

## Patients and methods

### Patients

Hundred-seventy three patients diagnosed with endometrial cancer, treated at the Radboud University Medical Centre, Nijmegen between 1999 and 2009, with an available preoperative serum sample were selected for this retrospective study. Preoperative diagnosis was based on diagnostic curettage or pipelle © endometrial sampling. Histology was performed by an expert gynecologic pathologist. All patients underwent an abdominal hysterectomy with bilateral salpingo-oophorectomy. Surgical staging with pelvic and/or para-aortic lymphadenectomy and/or omentectomy was performed in patients with a preoperatively high grade tumor and non-endometrioid histology. Patients diagnosed before 2009 were re-staged according the International Federation of Gynecology and Obstetrics (FIGO 2009) (Lewin 2011).

Adjuvant radiotherapy was applied according to the PORTEC criteria and chemotherapy was administered in patients with stage IIIC2 and IV disease (Creutzberg et al. 2000). Medical records of the patients were carefully reviewed.

Follow-up was performed from the date of primary treatment until the last visit or death. Follow-up visits were every 3 months during the first 2 years and subsequently every 6 months for the 3 years thereafter. After 5 years patients were dismissed from regular follow up. Blood samples were obtained by vena puncture and collected preoperatively in the outpatient clinic. The study was approved by the Ethics Committee of the Radboud University Medical Centre, Nijmegen.

### The aim

The primary aim of this study was to evaluate the correlation of concentrations of components of the PAS and VEGF in serum of patients with EC with known clinicopathological risk factors and outcome.

### Serum storage

Blood samples were obtained in dry tubes by vena puncture, centrifuged at 2000*g* during 10 min and serum was stored at − 40 °C until analyzed.

### Plasminogen activator system and VEGF measurements

Serum levels of the PAS components were determined by a enzyme-linked immunosorbent assays (ELISA). This procedure was described in detail by Grebenschikov et al. (1997). Prior to the assay samples were diluted; 160 times for PAI-1, 20 times for PAI-2 and 10 times for tPA and uPA. All measurements were performed in duplicate. In each run, international reference samples were run to check between-assay variability and to monitor overall performance of the assays (Grebenschikov et al. 1997; Sweep et al. 1998).

Antigen levels of VEGF in serum were measured by a specific ELISA as described by Span et al. (2000). All ELISAs applies a combination of four polyclonal antibodies (raised in four different animal species) employed in a sandwich assay format to exclude heterophilic antibody interference (Span et al. 2000).

### Statistical analysis

Statistical analysis was performed using GraphPad 5.3 (GraphPad Software, Inc, La Jolla, USA). In all tests *p* < 0.05 was considered to indicate statistical significance.

Serum levels VEGF, uPA, tPA and PAI-1 and -2, are presented as median values in ng/ml and log transformation was applied for a positively skewed distribution. Median serum levels were used as cut-off values to test differences between clinical subgroups. Clinical and pathological parameters were compared using the independent *t *test or Mann–Whitney *U* test, when appropriate. The Cox-proportional hazard model was used to assess the prognostic value of serum VEGF and components of the PAS both in univariate and multivariate analyses. VEGF and components of the PAS were used as log transformed median values. Traditional prognostic factors as FIGO stage, age, tumor grade, myometrial invasion and lymphovascular space invasion were included in a base model. VEGF and components of the PAS were entered separately in a second block. Points estimated were reported as hazard ratios (HR) and 95% confidence intervals (CI). In addition, Kaplan–Meier method was used to compute disease free and overall survival curves.

## Results

In total, preoperative serum samples of 173 patients with EC were examined. Clinical and pathological characteristics are presented in Table 1. Median age of all patients was 63 years (IQR 56–71). The majority of the patients were diagnosed with endometrioid type EC, 73% (*n* = 127). A substantial part of the cohort had an advanced stage of the disease, 35% (FIGO III/IV) and 65% had early stage disease (FIGO I/II).

**Table 1:** Demographic and tumor characteristics of all patients

	*N* = 173	%
Age		
Median, IQR	63 (56–71)	
BMI		
Median, IQR	27 (23–33)	
Histology		
Endometrioid	127	73
Non-Endometrioid	46	27
FIGO		
IA	17	10
IB	83	48
II	12	7
III	29	17
IV	32	18
Grade		
I	28	16
II	74	43
III	71	41
LVSI		
Yes	71	41
No	66	38
Missing	36	21
MI		
< 50	77	45
≥ 50	90	52
Missing	6	3
Lymph nodes		
Positive	14	8
Negative	48	28
Not assessed	111	64
Recurrence		
Locoregional	19	11
Distant	29	17
None	125	72

*MI *myometrial invasion,* LVSI *lymphovascular space invasion

Thirty-six percent of the patients had a lymph node dissection. Lymph node metastases were found in 23% (*n* = 14) of the patients who underwent lymphadenectomy during their primary surgery. In total forty-eight patients (28%) developed recurrent disease: 19 had locoregional and 29 had distant metastases.

### Correlation of VEGF and components of the PAS with clinical and pathological characteristics

Table 2 shows the correlation between serum levels VEGF and components of the PAS and various clinical and pathological factors. Median VEGF, uPA, tPA and PAI-1 and -2 concentration in all patients were 0.85 ng/ml (IQR 0.54–1.15), 3.49 ng/ml (IQR 2.40–5.83), 8.25 ng/ml (IQR 6.15–12.62), 180 ng/ml, (IQR 139–236) and 5.10 ng/ml (IQR 3.62–7.96), respectively.

**Table 2:** Clinicopathological factors in relation to serum levels

	VEGF	PAI-1	PAI-2	uPA	tPA
Age					
< 60	0.84 (0.54–1.14)	194 (135–248)	4.54 (3.53–5.98)	3.32 (2.09–5.94)	7.29 (5.88–10.36)
≥ 60	0.89 (0.56–1.28)	171 (142–231)	5.59 (3.69–8.48)	3.69 (2.47–5.86)	9.30 (6.36–13.73)
* p *value	0.31	0.69	**0.01**	0.22	**0.03**
FIGO					
I–II	0.88 (0.55–0.85)	171 (124–225)	4.73 (3.55–7.57)	3.32 (2.26–5.66)	7.85 (5.82–12.12)
III–IV	1.02( 0.58–1.35)	190 (150–261)	5.07 (3.62–7.40)	3.65 (2.52–5.25)	9.67 (6.42–12.87)
* p* value	0.32	**0.02**	0.89	0.95	0.13
Histology					
EC	0.83 (0.55–1.09)	175 (131–228)	4.66 (3.43–7.47)	3.41 (2.24–5.81)	7.85 (5.73–11.78)
Non-EC	1.02 (0.67–1.46)	186 (142–249)	5.49 (4.32–7.39)	3.34 (2.44–5.03)	9.66 (6.49–12.79)
*p* value	**0.03**	0.29	0.28	0.66	**0.05**
Grade					
I–II	0.81 (0.53–1.09)	172 (129–227)	4.54 (3.25–7.44)	3.30 (2.23–5.82)	7.55 (5.43–10.49)
III	1.00 (0.68–1.31)	189 (144–253)	5.57 (4.31–8.12)	3.49 (2.26–5.00)	9.66 (6.68–12.99)
* p* value	**0.02**	**0.04**	**0.01**	0.86	** < 0.01**
MI					
< 50%	0.82 (0.49–1.10)	183 (135–236)	4.58 (3.46–6.19)	3.40 (2.28–5.52)	8.67(5.84–12.14)
≥ 50%	0.95 (0.68–1.30)	181 (140–235)	5.97 (3.73–8.13)	3.50 (2.28–5.32)	8.29 (6.31–13.42)
* p* value	**0.02**	0.64	**0.04**	0.83	0.23
LVSI					
Yes	0.94 (0.68–1.18)	182 (140–250)	5.58 (3.92–8.12)	3.41 (2.02–4.80)	9.45 (6.39–13.35)
No	0.83 (0.53–1.16)	183 (124–230)	4.52 (3.41–5.97)	3.34 (2.24–6.27)	7.29 (5.31–10.35)
* p* value	0.15	0.25	**0.02**	0.16	**0.02**
Lymph nodes					
Positive	1.02 (0.61–1.21)	251 (190–315)	5.57 (4.53–7.44)	3.21 (2.77–5.29)	10.25 (6.43–14.49)
Negative	0.83 (0.50–1.15)	168 (117–225)	5.23 (3.59–8.38)	3.36 (2.01–4.92)	7.37 (5.95–10.37)
*p *value	0.18	** < 0.01**	0.35	0.45	0.08
Recurrence					
Yes	1.00 (0.66–1.29)	243 (169–315)	7.40 (5.00–9.59)	3.72 (2.88–4.99)	12.65 (9.66–17.15)
No	0.81 (0.53–1.09)	168 (124–214)	4.52 (3.25–6.15)	3.33 (2.13–6.53)	7.20 (5.70–10.07)
*p *value	**0.02**	** < 0.01**	** < 0.01**	0.49	** < 0.01**

Serum levels (ng/ml) are depicted as medians with 25th–75th percentile

Bold value indicates that the significant difference with a *p*-value < 0.05

*MI* myometrial invasion,* LVSI* lymphovascular space invasion

Serum VEGF and components of the PAS levels were significantly associated with stage of the disease, tumor histology, tumor grade, myometrial invasion, presence of lymphovascular space invasion (LVSI), lymph node metastases (LNM) and recurrence status Table 2. Preoperative serum levels of VEGF (1.00 vs. 0.81 ng/ml), PAI-1 (243 vs. 168 ng/ml) and -II (7.40 vs. 4.52 ng/ml) and tPA (12.65 vs. 7.20 ng/ml) were significantly higher in patients who developed recurrent disease compared to patients who remained disease-free.

PAI-1 serum levels were significantly higher in patients with advanced disease (190 vs. 171 ng/ml), high-grade tumors (189 vs. 172 ng/ml) and in patients with LNM (251 vs. 168 ng/ml). Both serum levels of PAI-2 and tPA were significantly associated with the presence of LVSI, higher tumor grade and age Table 2. Serum levels of uPA were not correlated with any of the clinicopathological factors. VEGF serum levels were significantly higher in case of MI, high-grade tumors and in non-endometrioid EC. VEGF serum levels were significantly higher in patients with local recurrences than distant recurrences (1.06 vs. 0.80 ng/ml, *p* 0.03). The other parameters didn’t correlate with the recurrence location.

### Survival analysis

Forty-eight of the 173 patients (28%) with EC developed a recurrence: 19 (40%) were locoregional and 29 (60%) were distant metastasis.

**Fig. 1:**
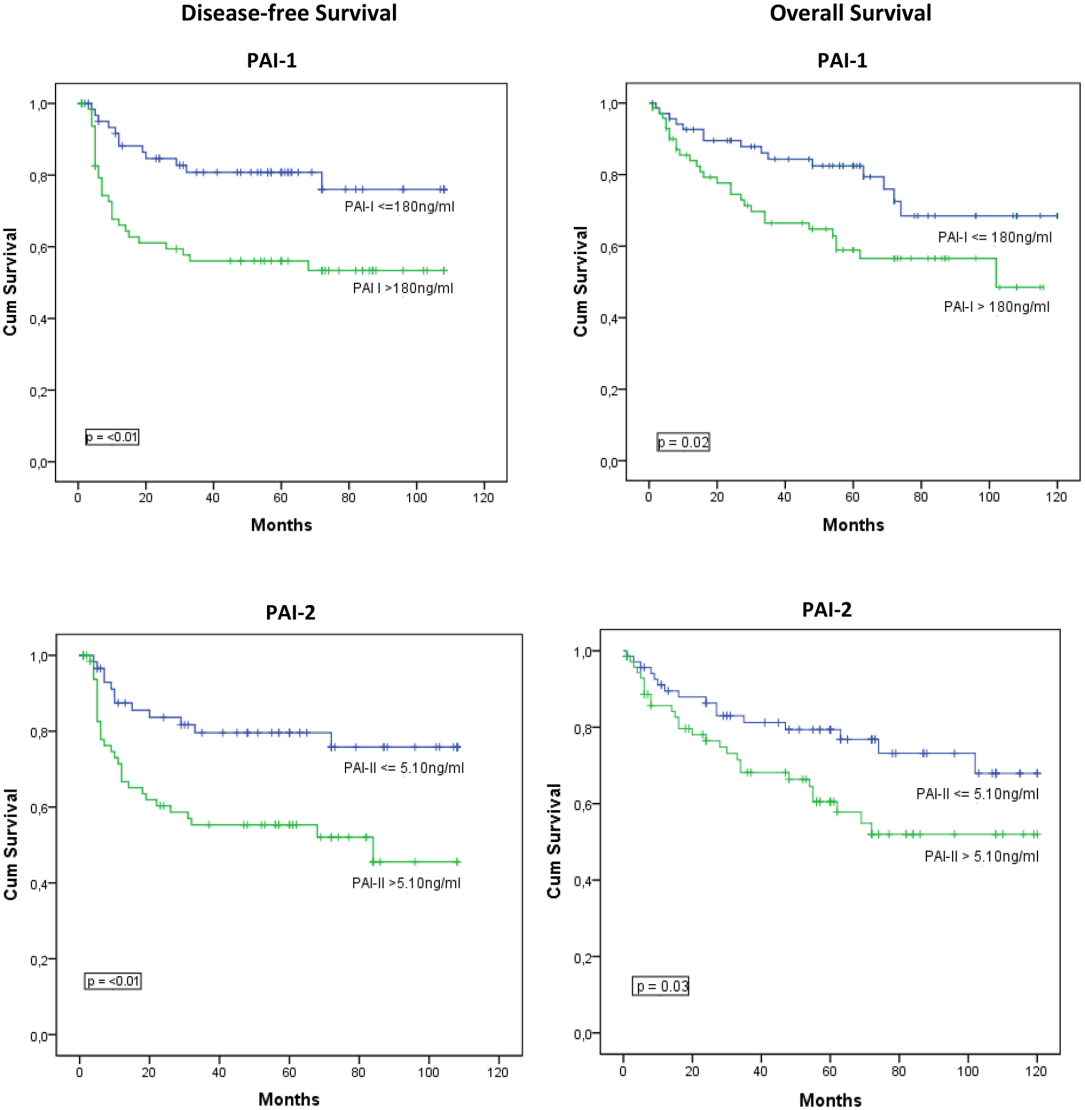

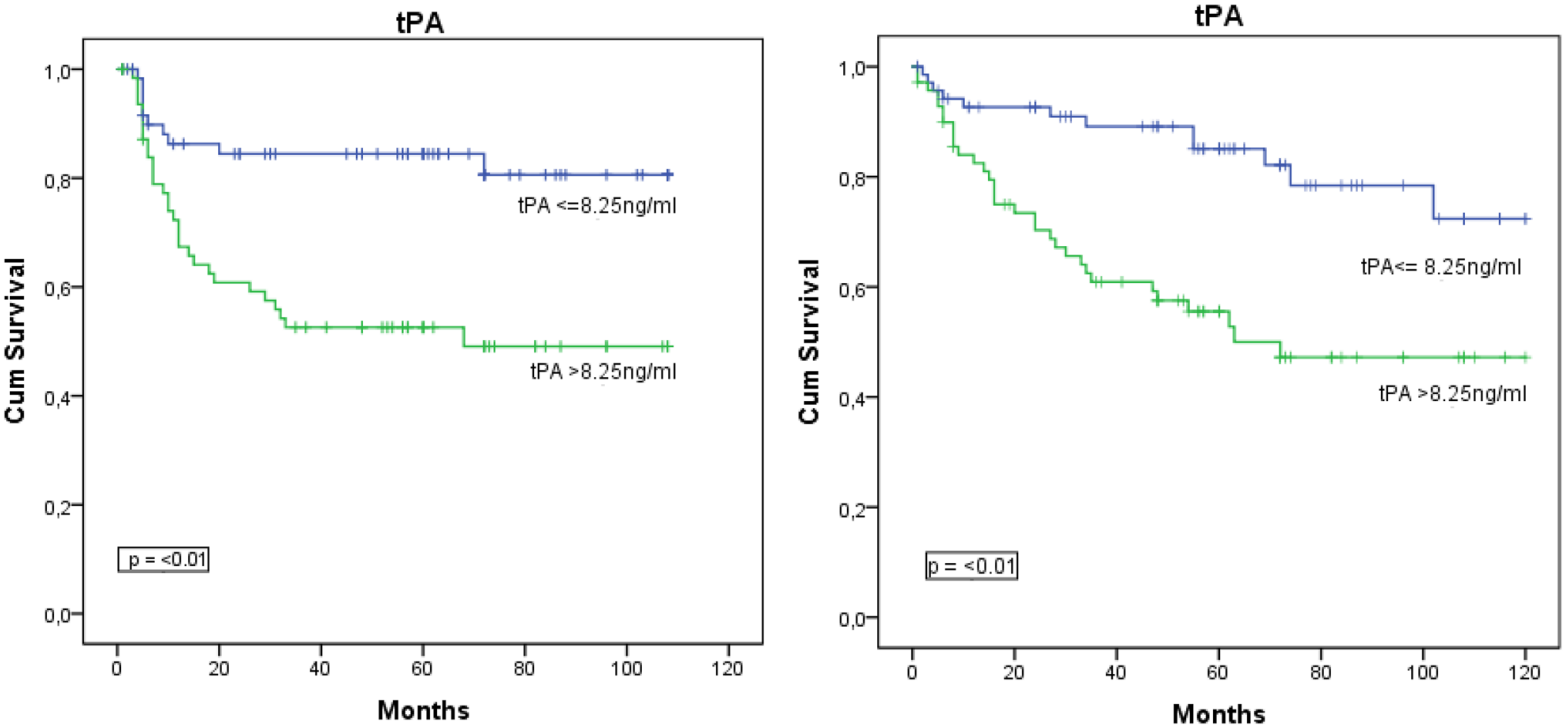
Kaplan–Meier curves for disease-free and overall survival as a function of PAI-1 and 2 and tPA. Median serum levels are depicted in the figures. Patients with > median serum levels of PAI-I, II and tPA had a significant shorter disease-free and overall survival

Kaplan–Meier curves were used to depict the disease-free survival (DFS) and overall survival (OS) in patients with ≤ median vs. > median serum levels of VEGF and PAS components. Figure 1 shows that patients with serum levels of PAI-1 and -2 and tPA above the median had a significantly worse DFS and OS than patients with serum levels below the median. No correlations were found between serum levels VEGF and uPA and DFS and OS.

In addition, we evaluated the prognostic relevance of VEGF and components of the PAS with univariate and multivariate survival analysis Table 3. Besides the classical prognostic factors including FIGO stage, age, tumor grade and LVSI, serum levels of PAI-1 and -2 and tPA were significantly associated with a reduced DFS and OS in the univariate analysis. Hazard ratios for DFS were 2.80 (95% CI 1.42–5.51), 2.94 (95% CI 1.50–5.74) and 3.57 (95% CI 1.74–7.30), respectively. Overall survival showed hazard ratios of 2.05 (95% CI 1.09–3.85), 1.94 (95% CI 1.05–3.60) and 3.23 (95% CI 1.66–6.29) for PAI-1 and -2 and tPA, respectively.

**Table 3:** Hazard ratios of overall survival and disease free survival

	Disease free survival survival					
	Univariate			Multivariate		
	HR	95% CI	*p *value	HR	95% CI	*p *value
A. Base model^a^						
FIGO stage						
III–IV vs. I–II	**5.09**	**2.84–9.09**	** < 0.01**	**4.30**	**2.12–8.70**	** < 0.01**
Age						
≥ 60 vs. < 60	**1.96**	**1.05–3.65**	**0.03**	**2.68**	**1.33–5.37**	** < 0.01**
Grade						
III vs. I–II	**2.45**	**1.38–4.33**	** < 0.01**	1.37	0.68–2.76	0.36
MI						
≥ 50% vs. < 50%	1.60	0.86–3.01	0.13	1.09	0.55–2.16	0.79
LVSI						
Yes vs. no	**2.30**	**1.23–4.30**	** < 0.01**	1.25	0.62–2.51	0.53
B. Additions to model (all continuous, log transformed, separately entered)						
Serum VEGF	1.52	0.81–2.85	0.19	1.83	0.91–3.69	0.08
Serum PAI-1	**2.80**	**1.42–5.51**	** < 0.01**	**3.85**	**1.84–8.07**	** < 0.01**
Serum PAI-2	**2.94**	**1.50–5.74**	** < 0.01**	**3.90**	**1.75–8.66**	** < 0.01**
Serum tPA	**3.57**	**1.74–7.30**	** < 0.01**	**2.53**	**1.16–5.55**	**0.02**
Serum uPA	1.67	0.89–3.12	0.10	1.65	0.83–3.28	0.14

	Overall survival					
	Univariate			Multivariate		
	HR	95% CI	*p *value	HR	95% CI	*p *value
A. Base model^a^						
FIGO stage						
III–IV vs. I–II	**5.75**	**3.19–10.34**	** < 0.01**	**3.47**	**1.71–7.06**	** < 0.01**
Age						
≥ 60 vs. < 60	**2.82**	**1.58–5.02**	** < 0.01**	1.91	0.97–3.77	0.059
Grade						
III vs. I–II	**3.42**	**1.92–6.10**	** < 0.01**	**2.37**	**1.18–4.78**	**0.01**
MI						
≥ 50% vs. < 50%	**2.57**	**1.29–5.09**	** < 0.01**	1.45	0.69–3.03	0.31
LVSI						
Yes vs. no	**2.08**	**1.10–3.94**	**0.02**	0.78	0.28–1.61	0.50
B. Additions to model (all continuous, log transformed, separately entered)						
Serum VEGF	1.47	0.79–2.77	0.22	1.37	0.68–2.75	0.37
Serum PAI-1	**2.05**	**1.09–3.85**	**0.02**	**2.09**	**1.08–4.05**	**0.02**
Serum PAI-2	**1.94**	**1.05–3.60**	**0.03**	1.99	0.99–3.99	0.05
Serum tPA	**3.23**	**1.66–6.29**	** < 0.01**	**2.16**	**1.06–4.44**	**0.03**
Serum uPA	1.30	0.71–2.39	0.38	1.04	0.54–1.99	0.89

Bold value indicates that the significant difference with a *p*-value < 0.05

*MI* myometrial invasion, *LVSI* lymphovascular space invasion

^a^The base model consisted of traditional prognostic factors (A), we separately entered the parameters in a second block (B)

The multivariate analysis adjusted for classical clinicopathological factors showed that PAI-1 and -2 and tPA were significantly independent prognostic factors for DFS with a HR of 3.85 (95% CI 1.84–8.07), 3.90 (95% CI 1.75–8.66) and 2.53 (95% CI 1.16–5.55), respectively.

Only PAI-1 and tPA turned out to be independent prognostic factors for OS, with a HR of 2.09 (95% CI 1.08–4.05) and 2.16 (95% CI 1.06–4.44), respectively.

## Discussion

This study showed that elevated serum levels of VEGF, PAI-1 and -2 and tPA in patients with EC were associated with known clinicopathological factors of poor prognosis and recurrent disease. PAI-1 and -2 and tPA were independent prognostic factors for DFS and OS in the multivariate Cox regression analysis.

The prognostic relevance of components of the plasminogen activator system has been intensively studied in various cancers (Duffy and Duggan 2004; Ferrier et al. 2000; Baluka et al. 2016). In breast cancer patients, high tissue levels of uPA and PAI-1 are associated with a poor prognosis, early disease relapse and predict treatment response and resistance (Foekens et al. 2000; Borstnar et al. 2002; Witte et al. 1999b; Gouri et al. 2016). High tissue levels of PAI-1 and uPA also led to a shorter DFS and OS in gastric, lung and ovarian cancer (Brungs et al. 2017; Su et al. 2015; Kuhn et al. 1999). These results are in line with the reported findings in EC tissue, where both uPA and PAI-1 tissue levels correlate with poor prognosis (Fredstorp-Lidebring et al. 1990; Tecimer et al. 2001; Dariusz et al. 2012; Steiner et al. 2008).

However, until now relatively little is known about the significance of the PAS in EC and to our knowledge no data is available of serum levels in relation with clinicopathological factors. Blood is easy to obtain and specific ELISAs are commercially available. It is a minimal invasive to acquire important prognostic information which could already be collected by the first clinical visit.

We found that high serum levels of PAI-1 were correlated with prognostic unfavorable factors such as; advanced disease stage, tumor grade and lymph node metastases (LNM). These results are generally in line with literature, where high PAI-1 tissue levels were associated with unfavorable prognostic factors in various cancer types (Brungs et al. 2017; Fredstorp-Lidebring et al. 1990; Tecimer et al. 2001; Steiner et al. 2008; Kohler et al. 1997; Lampelj et al. 2015). It might be expected that, based on its ability to inhibit uPA activity, PAI-1 would suppress cancer progression. However, consistent data suggest that PAI-1 is involved in mediating cancer progression, by enhancing angiogenesis, promoting tumor cell migration and blocking apoptosis and thus enhancing cell survival. (Duffy et al. 2014; Witte et al. 1999b; Manders et al. 2004a, b).

PAI-2, the other plasminogen activator inhibitor of the PAS is less frequently studied and its exact physiological function concerning carcinogenesis remains unclear. Under physiological conditions PAI-2 is not usually detectable in plasma, except during pregnancy when trophoblasts produce high levels of PAI-2 (Croucher et al. 2007; Verkleij-Hagoort et al. 2007). Decreased plasma levels of PAI-2 are correlated with preeclampsia and intrauterine growth retardation, so a role for the placental maintenance and fetal development is suggested.

Our cohort showed that high levels of PAI-2 were associated with MI, LVSI, higher tumor grade and age. However, we could not find a direct relationship with LNM. Literature concerning PAI-2 tissue levels in patients with breast, colon and gastric cancer showed the same results (Lewin 2011; Brungs et al. 2017; Su et al. 2015).

Tissue type plasminogen activator is known for its powerful capacity to remove fibrin deposits in the vascular system and is also involved in the carcinogenesis of different cancer types (Baluka et al. 2016; Ferrier et al. 1999; Borgfeldt et al. 2003). We found a significant correlation between elevated serum tPA and tumor grade and LVSI in our study which is in line with previously described results (Baluka et al. 2016; Borgfeldt et al. 2003).

Urokinase plasminogen activator has proven its prognostic relevance in breast cancer.

Interestingly, our results showed that serum levels of uPA did not correlate with any of the clinicopathological factors. In general, tissue levels of uPA do correlate with unfavorable prognostic factors in breast cancer as well as in gastroesophageal cancer and lead to a poor prognosis. On the other hand, the study of Grebenschtchikov et al. found that an association of uPA with clinicopathological factors was not reflected in plasma levels but only in tumor tissue of patients with breast cancer (Grebenchtchikov et al. 2005). Since, serum results of the PAS are scarce in both EC and other cancers, our results imply that the physiological function of the PAS regarding to carcinogenesis is different in tumor tissue compared to serum.

Literature concerning serum levels of VEGF in various cancer types (such as cervical cancer, colon, lung and ovarian cancer) report associations with clinicopathological factors and a poor prognosis (Zusterzeel et al. 2009; Komatsu et al. 2017; Kwon et al. 2010; Xuan et al. 2017). Our results are generally in line with these findings (Saarelainen et al. 2014; Dobrzycka et al. 2011; Gornall et al. 2001). We showed that serum levels VEGF were elevated in patients with non EC, MI and higher tumor grade. These findings were already described in patients with EC by Dobrzycka et al. and Saareleinen et al. (Saarelainen et al. 2014; Dobrzycka et al. 2011). In general, it is known that serum levels of VEGF are associated with a poor prognosis in different tumor types (Zusterzeel et al. 2009; Komatsu et al. 2017; Kwon et al. 2010; Xuan et al. 2017). However, we could not confirm these findings for EC.

Our results support the prognostic value of components of the PAS for DFS and OS which was found in EC tissue studies and other cancer types. (Borstnar et al. 2002; Borgfeldt et al. 2003; Nordengren et al. 2002). The DFS of patients with EC is shorter for those with elevated serum levels of PAI-1 and -2 and tPA. Besides this, patients with elevated serum levels of PAI-1 and tPA do also have a shorter OS.

Furthermore, PAI-2 turned out to be an independent prognostic factor for DFS. Unfortunately, no other studies are available reporting PAI-2 serum levels in relation with survival in patients with EC. However, our results are in agreement with those of Nordegren et al. (2002), who showed that high PAI-2 levels in tumor tissue EC patients were associated with a shorter DFS. Other studies concerning breast, lung and melanoma cancer report the finding that high tissue levels of PAI-2 leads to a favorable DFS and OS (Ferrier et al. 2000; Su et al. 2015; Yamashita et al. 1995; Foekens et al. 1995).

To date, the exact role of PAI-2 in carcinogenesis remains unclear. Studies have indicated that PAI-2 as well as PAI-1 may protect tumor cells against apoptosis (Nordengren et al. 2002). It seems that PAI-2 fulfills different physiological functions depending on tumor type as it is a predictor of both good and poor prognosis.

Our study showed that an elevated serum tPA level was associated with a reduced DFS and that tPA turned out to be the strongest prognostic factor for OS. TPA has a tumor metastasis promoting effect which causes plasmin mediated degradation of the extracellular matrix and facilitate local invasion and release of tumor cells into the circulation (Witte et al. 1999a; Borgfeldt et al. 2003; Kruithof and Dunoyer-Geindre 2014). Our results are in line with a study in ovarian cancer patients where elevated plasma tPA was significantly associated with a reduced OS (Borgfeldt et al. 2003). Although, de Witte el all reported that breast cancer patients with a high tPA tissue levels tended to have a better prognosis than those with low tissue levels (Witte et al. 1999a). This finding implies that tPA fulfills a different role in the carcinogenesis of breast cancer compared to EC and ovarian cancer.

Pre-operative blood is easy obtainable and could identify high risk carcinomas pre-operatively and subsequently individualize treatment.

PAI-1 and -2 and tPA have been implied to play a role in tumor growth, invasion and angiogenesis which is confirmed by the prognostic and predictive value in patients with EC (Tecimer et al. 2001; Dariusz et al. 2012; Nordengren et al. 2002). The combination of these three preoperative biomarkers may be valuable in the detection of high risk endometrial cancer and could identify a subpopulation who are at risk of recurrent disease.

This study is the first analyzing the value of preoperative serum levels of VEGF and components of the PAS, focusing on the prognostic and predictive value of these parameters in a well described cohort. Another strength is that pathology slides were reviewed by an expert gynecologic pathologist. However, some limitations need to be addressed. First, we included patients over a long period of time in which the FIGO stage classification and treatment guidelines have changed. We could only include patients of whom a serum sample was collected at the time of diagnoses and stored. Although, all available serum samples were included this could have led to selection bias. Serum of patients was stored over a long timeframe and possible influences on the VEGF and components of the PAS concentrations cannot be ruled out.

A future prospective study including a large cohort of patients could obviate these limitations and explore the preoperative value of serum VEGF and PAS components. As prediction of the clinical course is extremely important to prevent under and overtreatment and identify patients who are at risk of recurrent disease. VEGF and components of the PAS could lead to an adaption of the currently used predictive models which are still based on clinical and pathological features and subsequent individualize patients care and guide treatment options.

## Conclusion

This study shows that the PAS plays an important role in the carcinogenesis of endometrial cancer and is associated with well-known prognostic factors of poor prognosis. Preoperative sera of the PAS is easy obtainable and could identify patients who are at great risk of developing recurrent disease. Future studies should investigate if determinants of the PAS could be incorporated in a prognostic model and contribute to a more patient-personalized treatment plan.
